# Combination of Immunotherapy With Targeted Therapy: Theory and Practice in Metastatic Melanoma

**DOI:** 10.3389/fimmu.2019.00990

**Published:** 2019-05-07

**Authors:** Chune Yu, Xiaowei Liu, Jiqiao Yang, Min Zhang, Hongyu Jin, Xuelei Ma, Hubing Shi

**Affiliations:** ^1^Laboratory of Tumor Targeted and Immune Therapy, Clinical Research Center for Breast, State Key Laboratory of Biotherapy, West China Hospital, Sichuan University, Chengdu, China; ^2^Department of Liver Surgery, Liver Transplantation Center, West China Hospital, Sichuan University, Chengdu, China; ^3^Department of Biotherapy, Cancer Center, State Key Laboratory of Biotherapy, West China Hospital, Sichuan University, Chengdu, China

**Keywords:** targeted therapy, immunotherapy, combinatorial regimens, immune checkpoint blockade, metastatic melanoma

## Abstract

Metastatic melanoma is the most aggressive and obstinate skin cancer with poor prognosis. Variant novel applicable regimens have emerged during the past decades intensively, while the most profound approaches are oncogene-targeted therapy and T-lymphocyte mediated immunotherapy. Although targeted therapies generated remarkable and rapid clinical responses in the majority of patients, acquired resistance was developed promptly within months leading to tumor relapse. By contrast, immunotherapies elicited long-term tumor regression. However, the overall response rate was limited. In view of the above, either targeted therapy or immunotherapy cannot elicit durable clinical responses in large range of patients. Interestingly, the advantages and limitations of these regimens happened to be complementary. An increasing number of preclinical studies and clinical trials proved a synergistic antitumor effect with the combination of targeted therapy and immunotherapy, implying a promising prospect for the treatment of metastatic melanoma. In order to achieve a better therapeutic effectiveness and reduce toxicity in patients, great efforts need to be made to illuminate multifaceted interplay between targeted therapy and immunotherapy.

## Introduction

Skin cancer is one of the most common cancer types in the United States (1). Metastatic melanoma contributes to 90% the mortality of skin cancers, despite the fact that it accounts for only about 1% of all skin cancers (2). Melanoma is projected to be the fifth most common cancer in men and the sixth most common cancer in women in 2018. According to the American Cancer Society estimates, about 91,270 new cases and 9,320 deaths caused by melanoma are expected in 2018 (1). Although the incidence rates of melanoma increased over time, the mortality significantly declined in recent years. While early stage melanoma is usually curable with surgery, it becomes fatal once metastasis emerges. The 5-year survival for localized melanoma is 99%, which drops to 63 and 20% for melanoma with regional and distant metastasis, respectively (1). Therefore, the prognosis of metastatic melanoma is universally poor.

**Figure 1:**
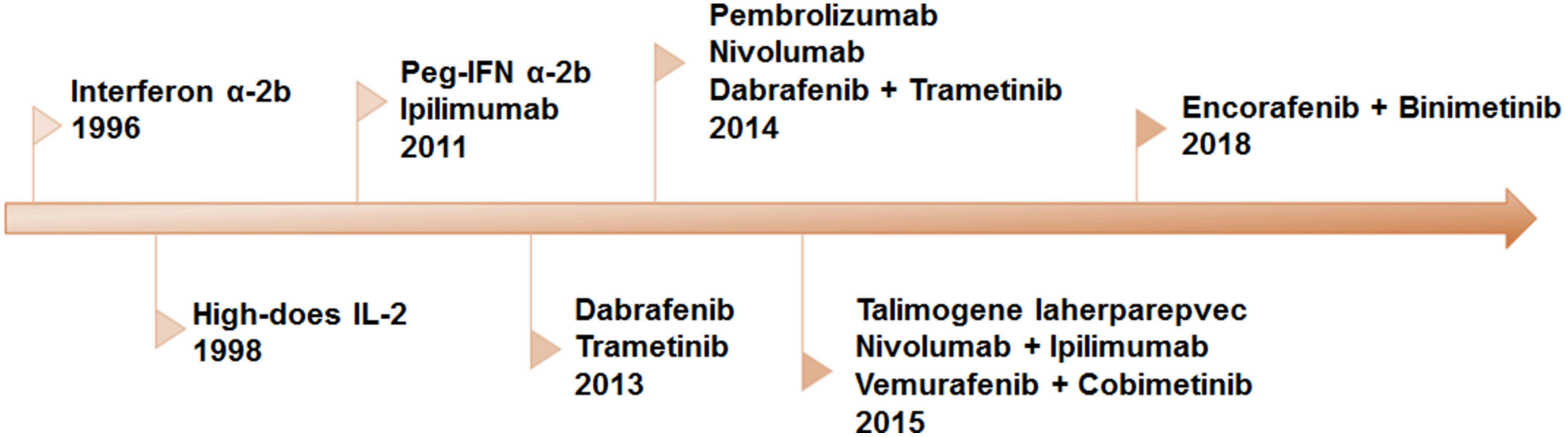
Timeline of FDA-approved regimens of targeted therapy and immunotherapy for melanoma. Interferon α-2b, IL-2, and peg-IFN were approved for adjuvant therapy. Since 2011, eleven therapies have been approved, including BRAF and MEK inhibitors as single-drug and combinatorial regimens, antibodies against CTLA-4 and PD-1 and the combination of the two.

Recently, significant progress in our understanding of the molecular mechanism of melanoma and the interaction between immune system and melanoma cells resulted in an amazing promotion in the development of novel therapeutic strategies. Over the past decades, eleven novel drugs, or combinatorial therapeutic regimens have been approved by FDA (Figure 1). All these regimens can be allocated into two categories: immunotherapy and targeted therapy. Long-term tumor remission can be achieved by immune checkpoint inhibitors, which is confirmed by improvements in overall survival (OS) and progression-free survival (PFS). However, the low response rate remains a predominant barrier to extensive benefits in clinical settings. On the contrary, the clinical responses to the targeted therapies are remarkable in the majority of patients with metastatic melanoma. Inspired by complementary advantages of these two regimens, a growing body of evidence indicated that combination of targeted therapy and immunotherapy may contribute to produce durable responses and minimize the toxicity in a broad spectrum of patients.

Here, we reviewed the current targeted therapies and immunotherapies of melanoma, discussed the rationality for the combination of above regimens. Moreover, we also summarized the results from preclinical studies and clinical trials of these combinatorial regimens for the treatment of metastatic melanoma and provided suggestions to achieve personalized therapy.

## Targeted Therapy In Metastatic Melanoma

In the past decades, a serial of aberrant oncogenic signaling pathways, have been identified that drive melanoma progression (3–5). Among these pathways, mitogen-activated protein kinases (MAPK) cascades consist of three liner kinases RAF/MEK/ERK, and play a pivotal role in the malignant transition of melanoma (6). About 70% of cutaneous melanoma carries genetic mutations or dysregulations of the kinases in this pathway, e.g., BRAF or NRAS mutations as well as loss of NF1 (7–11). Small molecule inhibitors or antibodies blocking the oncogenic cascades were designed and developed (12), with several agents been approved by FDA for the treatment of metastatic melanoma (Figure 1). Currently, our discussion primarily focuses on the classic BRAF inhibitors and MEK inhibitors. Additionally, small molecules targeting other oncogenes have been comprehensively summarized by several excellent review articles (13, 14).

### BRAF Targeted Therapy

BRAF (serine/threonine protein kinase), a member of RAF family kinases, is an integral part of MAPK pathway (15). About 50 to 60% of cutaneous melanomas express an activating mutation in BRAF^V600^, in most cases (more than 90%), a substitution of a valine for a glutamic acid was observed at the codon 600 (V600E) (16). BRAF mutant causes continuous activation of MAPK pathway and leads to uncontrolled proliferation of tumor cells with multiple mechanisms, making itself an attractive therapeutic target (17, 18).

To date, three drugs targeting the BRAF^V600E^ mutation have been approved for the treatment of metastatic melanoma patients with BRAF^V600E^ mutation, namely vemurafenib, dabrafenib, and encorafenib (19–22). In 2011, vemurafenib (PLX4032) became the first BRAF^V600E^-targeted drug approved by FDA. In a latest phase III trial (NCT01006980), vemurafenib significantly improved OS (84 vs. 64% at 6 months) and response rate (48 vs. 5%) compared with standard chemotherapy (20). Dabrafenib, was another BRAF^V600E^ inhibitor approved by FDA in 2013. Similar to vemurafenib, dabrafenib produced higher response rates and improved OS and PFS than dacarbazine. In June 2018, encorafenib was approved by FDA in combination with binimetinib. Although BRAF targeted therapy achieved a remarkable clinical response in patients with BRAF^V600E^, acquired resistance was developed promptly after initial response. The median PFS was usually observed within 5 to 7 months (20, 23, 24). In addition, a part of melanoma patients with BRAF^V600E^ mutation do not respond to BRAF inhibitors, which was regarded as primary resistance (9, 20, 25). Several mechanisms of primary and acquired resistance have been verified over the past decades, and some of them have been clinically validated (9, 26–29).

### MEK Targeted Therapy

In the meantime, a serial of MEK1/2 inhibitors, including trametinib, cobimetinib, and binimetinib were approved by FDA as either monotherapy or combinatorial therapy for the treatment of metastatic melanoma (30–32). As the first approved MEK inhibitor, trametinib significantly improved OS (81 vs. 67%) of patients with BRAF^V600E^ mutation compared with chemotherapy (33). After being approved by FDA in 2013 as a monotherapy, trametinib was further approved as a combinatorial regimen in the following year. Without additional overall toxicity, the median PFS prolonged from 5.8 months as single-agent to 9.4 months with combination of trametinib and dabrafenib (34). Cobimetinib, another MEK inhibitor developed by Exelixis and Genentech (Roche), had an encouraging performance in unresectable and metastatic BRAF^V600E^-mutant melanoma when applied in combination with vemurafenib. Such regimen significantly improved OS and PFS compared with vemurafenib monotherapy with severe adverse effects (35). Based on the results of COLUMBUS trial, a phase III study of 577 patients with metastatic or unresectable melanoma harboring BRAF^V600E^ mutation, the combination of encorafenib and binimetinib (a BRAF inhibitor) was approved by FAD in June 2018. As reported, median overall survival (33.6 vs. 16.9 months) and media PFS (14.9 vs. 7.3 months) were significantly improved in encorafenib plus binimetinib group comparing with vemurafenib group (36). The combination of encorafenib and binimetinib is the first targeted therapy which led the overall survival to more than 30 months in melanoma patients with BRAF^V600E^ mutation in phase III clinical trial (32, 36). Hence, it sets a new standard for the management of BRAF^V600E^-mutant melanoma ever since.

Although targeted therapies, especially the blockades of multiple targets lead to impressive and rapid tumor regression in the majority of patients with BRAF^V600E/K^ mutation, the responses are always not durable due to acquired resistance. The resistant mechanisms have been intensively investigated and the potential secondary aberrances fall into two categories. One is related to MAPK pathway reactivation, including: (1) mutation of NRAS^Q61R/K^ in the upstream of BARF (9); (2) loss of RAS suppressor gene NF1, which leads to the activation of MEK (10); (3) up-regulation of COT1 and protein aggregation (37); (4) selective amplification of oncogenetic BRAF^V600E^ mutation, and subsequent increase the transcriptional level and protein expression of BRAF mutants (38); (5) change in splice variants at the RNA level, leading to selective partial loss of the exon sequence in BRAF^V600E^, thus forming a RAS-independent, continuously activated monomer (27); (6) mutation of the MEK1/2 gene (26). Another is related to the activation of alternative oncogenic pathways, including: (1) up-regulation of PDGFRβ (9, 39); (2) up-regulation of c-MET, as well as its ligand HGF (40, 41); (3) up-regulation of IGF1R (25); (4) mutations of PI3K subunits (42, 43) (gain-of-function mutation of PIK3CA/PIK3CG and loss-of-function mutation of PIK3R2); (5) gain-of-function mutation of AKT1/3 (42, 44); (6) deletion or inactivating mutations of the PTEN gene (42, 45, 46). Although blockade of secondary pathways via rational combinatorial therapy partially delayed the occurrence of tumor relapse, the resistant mechanisms will be developed ultimately. Therefore, an alternative approach with distinct principle is highly desired.

## Immunotherapy

Immunotherapy is another major breakthrough in the treatment of metastatic melanoma. So far, seven drugs have been approved and they all showed long-term tumor inhibition (Figure 1). By definition, immunotherapy utilizes the host's immune system to eliminate tumor by sensitizing patient's intrinsic immune system or strengthening the previous response and thus achieves durable response. T-cell mediated therapy was regarded as the most powerful approach among all immunotherapeutic regimens. The generation of antitumoral T-cell response is complex. It involves a variety of immune cells and multiple steps which include augmenting tumor-associated antigens (TAAs) presentation, T cell priming and differentiation, enhancing the activity of T cells, and overcoming the immune suppressive signaling (Figure 4). The augmentation of tumor immunotherapy can be summarized into three major manners: (1) non-specific immunomodulation aiming to eliminate tumor by stimulating effector T cells (cytokines: IL-2, IFN α-2b), activation of stimulatory molecules and blockade of inhibitory molecules which prevents the activation of T cells (PD-1, CTLA-4); (2) activation of specific immune response including tumor vaccine and oncolytic virus; (3) adoptive immunotherapy or cell transfer which passively transfer a large number of activated T cells for the tumor infiltrating lymphocytes (TILs) (ACT, TCR-engineered T cell, and CAR-T) (48). In chronological sequence, T-cell mediated immunotherapy experienced 4 generations, including vaccine, cytokines, adoptive T cell transfer, and immune checkpoint blockade. Here, we are going to do a brief retrospect in inverse order.

### Immune Checkpoint Blockade

Immune checkpoints are composed of various inhibitory molecules that act as homeostatic regulators of the immune system that are critical for maintaining self-tolerance as well as diminishing excessive systemic inflammation in the host (49). However, it is now clear that tumors can hijack these inhibitory mechanisms in the tumor microenvironment to evade the immune destruction and lead to the recurrence of malignant melanoma eventually. So far, two immune checkpoint blockades, cytotoxic T lymphocyte-associated 4 molecule (CTLA-4) and programmed death 1 (PD-1) have been intensively studied in the treatment of metastatic melanoma. Monoclonal antibodies designed to block CTLA-4 and PD-1 reactivate antitumor immune responses and result in remarkable clinical benefits. However, it is important to emphasize that other major immune checkpoint blockades may be potential therapeutic strategies and some clinical trials of them are underway.

#### CTLA-4 Blockade

CD28 is a co-stimulatory molecule expressing on the surface of T cells. Upon binding to B7-1 and B7-2 on antigen-presenting cells (APCs), CD28 trans-activates TCR signaling and thus improves antitumor response when antigen recognition occurs (50). However, CTLA-4, one of the first negative regulators to maintain T cell activation response, has higher affinity to compete with CD28 for binding to its ligands and induces immunosuppression. In addition to limiting CD28 downstream signaling mediated by PI3K/AKT (51), the immunoreceptor tyrosine-based inhibitory motif (ITIM), the cytoplasmic domain of CTLA-4, will recruit SHP family phosphatases which lead to reverse phosphorylation of signaling molecules by TCR activation. Moreover, upregulated expression of CLTA-4 on Tregs (CD4^+^ FOXP3^+^) is pivotal to their immunosuppression function (52). Specific deletion of CTLA-4 in Tregs is sufficient to maintain aberrant T-cell activation and lead to autoimmunity (53).

CTLA-4 has become a charming target for the treatment of melanoma aiming to strengthen effector T cell function and achieve durable response based on the negatively regulated T cell activation. Monoclonal antibodies which specifically block CTLA-4, enhance effector T cells function and inhibit Tregs-associated immunosuppression have been demonstrated in murine models and melanoma patients. Ipilimumab, a fully human monoclonal antibody IgG1 that inhibits the interaction between CTLA-4 and its ligands, was approved by FDA in 2011 for the treatment of unresectable stage III/IV melanoma, owing to the improvements in clinical outcomes. In a randomized phase III trial, the OR was significantly improved in patients with ipilimumab plus gp100 vaccine compared with vaccine alone (10 vs. 6.4 months). No difference in OS was observed between ipilimumab alone and ipilimumab plus gp100 vaccine (54). However, more grade 3/4 immune-related adverse effects were observed in ipilimumab than vaccine group (15 vs. 3%), because of the continuously activated T cells. Moreover, only 10% of advanced melanoma patients acquired clear, objective responses, though the responses were durable. Therefore, it is urgent to explore a way to improve the clinical response and identify the biomarkers of CTLA-4. Previous studies indicated that clinical benefits were significantly associated with neo-antigen load, overall mutational load, and the expression of cytolytic markers in the immune microenvironment (55). Interestingly, a recent research proved that CTLA-4 is also expressed on melanocytes and melanoma cells and it was regulated by IFN-γ, which is a potential strategy to cure melanoma (56). Nowadays, combinatorial therapy has become a strong scientific rational and efficient regimen for the treatment of melanoma, such as combination with targeted therapy, chemotherapy or other immunotherapies. Some clinical trials of these regimens are undergoing (NCT00803374, NCT02743819, and NCT01721746). Tremelimumab, another monoclonal antibody directly targets CTLA-4, is still in clinical development since it failed to demonstrate advantages in survival compared with standard chemotherapy in a phase III trial, even though tremelimumab generated durable tumor regression in phase I and II trials (57). It has been verified in the latest studies that ipilimumab eliminates tumor cells via the depletion of Tregs in tumor microenvironment rather than immune checkpoint blockade and this mechanism depends on Fc receptor on host cells (58).

#### PD-1/PD-L1 Blockade

Programmed cell death protein 1 (PD-1) is an important negative regulator of T cell activity. Interacting with two cell surface ligands, PD-L1 (CD274) and PD-L2 (CD273) expressing on immune cells or tumor cells, it inhibits T cell activation. The mechanism of the suppressive effects of PD-1 involves simultaneous pro-apoptotic effect in cytotoxic T cells and anti-apoptotic effect in Tregs. The binding of PD-1 and its ligands leads to the damage of T cell activation and function regulated by recruiting phosphatase src homology 2 (SHP-2), which dephosphorylates signaling elements, and downregulating TCR signaling by inactivation of Zap70 (59, 60). Nevertheless, co-stimulatory signaling (CD28) played a vital role in TCR signaling responding to PD-1/PD-L1 axis according to a recent study. This study utilized a biochemical reconstitution system and demonstrated that PD-1/PD-L1 interaction resulted in preferential dephosphorization of CD28, but not TCR by recruiting SHP-2 (61). Distinctively, Rota et al. believed that SHP-2 was not crucial for dysfunctional response of exhausted T cells for PD-1 signaling pathway *in vivo* (62). These findings indicated downstream pathways of PD-1 are functionally redundant, which was possibly implemented by redundant phosphatases. Normally, this negative feedback mechanism of PD-1/PD-L1 axis balances the immunity and immunopathology, thus to diminish tissue damage while limiting anti-tumor activity through immune evasion. PD-1 is always highly expressed on activated or exhausted T cells subsequent to persistent exposure to high antigen loads. Typically, PD-L1 is upregulated on APCs or tumor cells which are capable of evading immune system surveillance, including metastatic melanoma cells (63, 64). PD-L1 is expressed on various cell types including T cells, B cells, NK cells, and tumor cells, the expression of which is driven by cytokines (IFN-γ) dependent and independent mechanisms, and the latter involves PTEN deletion, anaplastic lymphoma kinase (ALK) and EGFR mutation (65–67). Sometimes, the expression of PD-L1 is a biomarker for immunotherapy, whereas the expression of PD-L2 is largely confined to APCs. In addition to inhibiting the activation and other functions of T cells, PD-1 signaling may also regulate metabolic reprogramming, attenuate glycolysis and simultaneously promote lipid catabolism and fatty-acid oxidation, induce energy derivation, and partly lead to T cell exhaustion (68). PD-1 is a marker of effector T cells because it is expressed on all of the activated T cells, but not an exhaustion-specific molecule. PD-1 blockade can increase tumor rejection by reinvigorating T cell function, making it a predominant target for immunotherapy.

It was another breakthrough of immune checkpoint blockade that nivolumab (BMS-936558) and pembrolizumab, two fully human anti-PD-1 monoclonal antibodies, were approved by FDA for the treatment of unresectable or metastatic melanoma in 2014. In a phase III trial, nivolumab dramatically improved PFS (5.1 vs. 2.2 months) and OS at 1 year (72.9 vs. 42.1%) compared with dacarbazine in metastatic melanoma without BRAF mutation. Besides, grade 3/4 adverse events were lower in nivolumab group (11.7%) than in dacarbazine group (17.6%) (69). As reported, drug-related adverse events with nivolumab were lower than those with ipilimumab (70). Similarly, pembrolizumab had better results in clinical outcomes than ipilimumab in advanced melanoma (71). Despite the dramatic progress in prognosis with monotherapy of PD-1 blockade, remission sustained only in a subset of patients. Therefore, it is crucial to selectively target this population and develop effective combinatorial strategies for patients not benefiting from monotherapy. The expression of PD-L1 in tumors may be an indicator for the prognosis (72, 73). Other parameters have also been mentioned, such as: (1) genetic signatures enrichment (metabolic signatures, mesenchymal, and suppressive inflammatory transcriptional phenotypes); (2) the existence and activity of TILs (more clonal T cell population and less TCR diversity, transcriptional signature in which cytokine genes are increased); (3) general immune status of the patients (neutrophil to lymphocyte ratio and the frequency of circulating monocytes); (4) “tumor foreignness” (MSI-H tumors carry high mutational load; neoantigens); (5) the presence of other inhibitory signaling within tumor cells (MDSCs, Tregs, inhibitory molecules) (74). Additionally, gut microbiome might regulate the response to PD-1 blockade immunotherapy in melanoma patients. More specifically, enrichment of *Ruminococcaceae* family in gastrointestinal system is associated with a better prognosis (75). In order to maximize the clinical outcomes, combinatorial therapy is in need to further strengthen antitumor efficacy. Combination of anti-PD-1 with anti-CTLA-4 therapies significantly induced tumor regression in various cancer types, including melanoma. According to a recent clinical trial, for PD-L1-positive melanoma patients with brain metastasis who received nivolumab plus ipilimumab, the intracranial clinical benefit rate was 57%, objective response rate was 55%, complete response rate was 26%, with 6-, 9-, and 12-months survival of 92.3, 82.8, and 81.5%, respectively. Additionally, the incidence of immunotherapy-related adverse effects was not different from that of nivolumab or ipilimumab alone (76, 77). In addition to PD-1 blockade, anti-PD-L1 antibody has also been verified as an effective approach to improve antitumor effect by disrupting PD-1 signaling. As shown by Wang et al. the combination of diprovocim (TLR1/TLR2 agonist) and anti-PD-L1 eliminated melanoma completely in mice model by increasing TILs (78).

#### Other Immune Checkpoint Blockades

Apart from CTLA-4 and PD-L1, other immune checkpoints expressed on activated or exhausted T cells include LAG-3, TIM-3, TIGIT, CD96, BTLA and CD160, which dampen T-cell effector function via diverse inhibitory signaling pathways. LAG-3 is similar to CD4 co-receptor in structure with greater affinity to MHC class II than CD4 (79). In addition to expression on activated T cells, LAG-3 was also found on the surface of NK cells (80), Tregs (81), as well as plasmacytoid dendritic cells (DCs) (82). As an immune inhibitory regulator, LAG-3 has the potential to limit autoimmunity but it may also impair the ability to eliminate tumor cells or pathogens through attenuating T cell proliferation and activation and instructing Tregs in their suppressive activity. While the antibody against LAG-3 treatment alone has little impact on restoration of T cell function, the combination of PD-1/PD-L1 and LAG-3 co-blockade can remarkably improve T cell activation (83, 84). It might be possibly explained that LAG-3 and PD-1 co-expressing on TILs provides a promising combinatorial approach to enhance antitumor activity (83). Combination of anti-PD-1 antibody and LAG-3 for the treatment of patients with stage III/IV melanoma is currently undergoing (NCT02676869). TIM-3 is selectively expressed on FoxP3^+^CD4^+^ T helper and IFN-γ producing CD8^+^ cytotoxic T cells. It impairs the function of effector T cells and promotes immunological tolerance when binding to its ligands such as galectin-9 and HMGB1 (85, 86). TIM-3 is also expressed on DCs and inhibits DCs function through blockading NF-κB. The upregulation of TIM-3 on NK cells is associated with a poorer prognosis (87). Interestingly, TIM-3-deficient mice did not experience autoimmunity, which suggested that targeting TIM-3 will be unlikely to produce adverse effects (88). INCAGN02390, a novel antagonist against TIM-3 is undergoing trial for the treatment of advanced malignancies including melanoma (NCT03652077). Other clinical trials are being carried out in order to test the efficacy of combinatorial regimens with PD-1 blockade (NCT02817633, NCT03058289, and NCT02608268). TIGIT and CD96 may compete with stimulatory regulator CD226 for binding to nectin and nectin-like proteins (89). Therefore, it plays an important role in lymphocyte-mediated effector functions of tumor regression.

#### The Supplementary of Immunosuppressive Regulators

Tryptophan-2,3-dioxygenase (TDO), indoleamine 2,3-dioxygenase 1 (IDO1), and indoleamine 2,3-dioxygenase 2 (IDO2) play pivotal roles in the catabolism of tryptophan and other indole compounds which are important to maintain the proliferation and function of immune cells. Degradation of tryptophan leads to the generation and maintenance of immunosuppressive microenvironment, which may contribute to immune escape in cancers (90). High expression of IDO and TDO were related to a poor prognosis in several types of cancers (91). IDO upregulation leads to immune evasion by directly attenuating T cell function, promoting differentiation of Tregs, decreasing DCs activities, recruiting and activating MDSCs (92, 93). Thus, it is a promising therapeutic strategy to inhibit IDO. Excitingly, IDO inhibitors and CTLA-4 blockade synergize intensively to promote tumor rejection *in vivo* through inhibition of immunosuppressive environment and activation of intratumoral T cells (94). Several clinical trials are ongoing to evaluate the effects of IDO inhibition combined with vaccine (NCT01961115) or immune checkpoint blockade (NCT02073123). IDO-peptide vaccine is a novel therapeutic approach. Specifically, IDO-specific T cells could recognize and kill IDO-positive tumor cells and induce IDO-specific memory T cells (95). IDO/PD-L1 peptide vaccine in combination with nivolumab for the treatment of patients with metastatic melanoma is under clinical validation (NCT03047928). Regrettably, a phase III trial showed that the combination of epacadostat (an IDO inhibitor) with PD-1 blockade failed in improving PFS compared with PD-1 blockade alone in advanced melanoma. Nevertheless, lessons can be learned as previously proposed, possible explanation might be related with improper dosage, inappropriate regimen of combination, acquired resistance to IDO inhibitor and/or IDO/TDO dual inhibitor, as well as blockade of AHR pathway (96).

### Adoptive T Cell Transfer, TCR-Engineered T Cell, and CAR-T

In adoptive T cell transfer (ACT) therapy, patients are infused with a large amount of autologous reactive and tumor-specific T cells expanded *ex vivo* accompanied by IL-2 administration. These T cells are isolated from either peripheral blood or the tumor tissue. Impressive regressions of cancers in advanced or metastatic stage were reported with this method. In addition to the natural host tumor-specific T cells, antitumor activity can be exhibited by genetically modification with chimeric antigen receptors (CARs) or antitumor T cell receptors (TCRs). TIL is a form of tumor-specific T cell, originating from tumor tissues with broad-spectrum heterogeneity. The ability to kill tumor cells is weakened when isolated from tumor tissue. However, it can be strengthened by IL-2 stimulation (97). Before the cell infusion, host lymphodepletion should be prepared. Particular attention should be paid during the process. First, the dose of IL-2 and the extent of lymphodepletion should be cautiously determined. Second, TILs are prepared as a mixture of CD4^+^ and CD8^+^ T cells, since both of them contribute to tumor regression. Third, TILs prefer recognizing neo-antigens derived from mutations and much effort should be devoted to screening for the neo-antigens and expanding of mutation-specific T cells, despite the methodological challenges. The differentiation status of T cells is also a key factor to strengthen the antitumor activity. Another type of tumor-specific T cells is derived from peripheral blood. The stimulation by conjugating peptide antigen is necessary to obtain the desired T cells specificity. Tumor-specific antigens may either be exclusively expressed in tumor tissue or higher expressed in tumor tissue than in normal tissue. However, the latter will lead to on-targeted and off-tumor toxicity such as targeting the melanocyte antigens Melan-A (MART-1) or gp100 which may trigger autoimmune attack of normal tissue with melanocytes, such as skin, eye, and ear. Therefore, looking for antigens uniquely expressed in tumor is a direction to improve the effectiveness of ACT. A recent search suggested that antitumor-specific T cells may be the populations of PD-1^+^CD8^+^ lymphocytes from peripheral blood (98).

Chimeric antigen receptor T cell therapy (CAR-T) is a novel immunotherapy for cancers. It involves adoptive cells with are genetically engineered with a chimeric antigen receptor to target tumor specifically. First-generation CARs were comprised of CD3ζ or FcRg, which is a single intracellular signaling domain combined to the transmembrane. Because of the absence of co-stimulatory molecules such as CD28, CD27, 4-1BB, or OX-40, the outcomes of patients were unsatisfactory (99). To further improve the antitumor effectiveness, second- and third- generation CARs attempt to incorporate one or more co-stimulatory domains into a single CD3ζ-based cytoplasmic domain. In order to diminish the toxicity, fourth-generation CARs transduced cytokines (IL-12) fragments and are capable to release IL-12 to the tumor environment consistently, which is named TRUCK T cell. This modification resulted in an impressive improvement in CAR-T cell persistency, effector function, and rapid elimination of tumor cells. In a recent phase I-IIa study, the overall remission rate was 81% at 3 months and overall survival was 90% with anti-CD19 CAR T-cell therapy in children and young adults with B-cell lymphoblastic leukemia. However, grade 3/4 adverse effects occurred in 73% patients, mainly the cytokine release syndrome (CRS) and neurotoxicity (100). CRS was caused by the amount of cytokines and chemokines released by activated CAR T cells or other immune cells. The hallmark of it is featured with elevated level of IL-6 and can be managed with tocilizumab, an IL-6 receptor antagonist. The latest studies verified that CRS was mediated by IL-1 and IL-6 produced by macrophages and can be abolished by IL-1 blockade (101). Fraietta et al. found that CD27^+^PD-1^−^CD8^+^ CAR T cells with higher expression of IL-6 receptor is a biomarker of CAR-T therapy and is responsible for tumor control (102). Although CAR-T therapy has been successful in hematological malignancies, less response was seen in the treatment of solid tumors such as melanoma. Lack of proper tumor associated antigens, poor T cell infiltration in tumor, and unfavorable immunosuppressive tumor microenvironment may be the possible reasons for this failure (103). Combination of CAR-T therapy and immune checkpoint blockade, targeted therapy might induce desired clinical responses.

TCR-engineered T cell is the second genetically engineered adoptive T cell with engineered TCR gene that is comprised of TCR α and β subunits and has high-affinity to recognizing and binding antigen peptides which are represented by MHC on the surface of APCs. MART-1 and gp100 TCR gene-engineered T cells improved the regression of tumor, meanwhile the toxicities occurred in eyes, ears and skin (104). NY-ESO1, a cancer-testis antigen, is exclusively expressed on the germline tissue and may reduce the toxicity of on-targeted off-tumor. In melanoma patients, NY-ESO1 engineered TCR T cell significantly improved the objective response rate (105). Other cancer-testis antigens including MAGE family presented in the context of HLA or other MHC I subclass, are also targeted by engineered TCR T cells. Similar to MART-1, both improvement of response and toxicities were achieved. Therefore, the specific antigens such as neoantigens and combinatorial strategies need to be further exploited.

### Cytokines

Cytokine is a traditional immune enhancement approach that can promote tumor regression and induce durable clinical response. To date, IL-2 and IFN-α are the only FDA-approved cytokines as adjuvant therapeutic agents for the treatment of melanoma, although other cytokines (IL-12, IL-15, IL-18, IL-21, and GM-CSF) have shown profound results in clinical settings (106, 107). IL-2 activates B cells and NK cells and high-doses of IL-2 induces durable complete responses. Based on the facts above, it became the first FDA-approved immunotherapeutic agent for the treatment of metastatic melanoma in 1998. However, treatment of high-dose IL-2 resulted in severe adverse events, such as reversible multisystem organ failure, peripheral edema, seizures, and breathing problems (108). Surprisingly, a recent meta-analysis showed that rates of complete response were similar with high and intermediate dose of IL-2 for the treatment of melanoma. Therefore, the therapeutic dose should be re-considered (109). Additionally, orthogonal IL-2 cytokine-receptor complexes that specifically target engineered T cells may lower off-target effects and adverse effects, which might be a novel strategy to cure cancers (110). Previous researches proved that CTLA-4 and PD-1 negatively regulated T cell function via IL-2 dependent and independent mechanisms, which provided a theoretical support for combinatorial strategy (111, 112). Preclinical studies confirmed that combination of IL-2 and TNF-α armed virus and anti-PD-1 therapy was a promising regimen and a clinical trial has been scheduled (113). IL-2 in combination with targeted therapy (NCT01603212), immunotherapy (NCT03476174, NCT01659151), chemotherapy (NCT01124734) and radiotherapy (NCT01416831) are included in the clinical trials.

Interferon alfa-2b (IFN α-2b) plays a significant role in the antiangiogenic, immunomodulatory, and antitumor activities and may activate T cells, B cells, NK cells, and DCs. IFN α-2b was approved by FDA in 1996 as an adjuvant treatment for patients with resected stage IIB/III melanoma due to the improvement in RFS and OS (114). Recently, a meta-analysis suggested that RFS can act as a surrogate for OS for adjuvant treatment in the patients with high-risk stage II-III melanoma (115). Pegylated interferon-alfa 2b (Peg-IFN), INFα-2b covalently coupled with polyethylene glycol (Peg) that prolongs its circulation time in the blood, was approved by FDA in 2011 for the treatment of node-positive resected melanoma as an adjuvant therapy. Though advanced peg-IFN therapy significantly improved PFS and OS, a case of malignant melanoma developed multiple metastases after switching from IFN b to peg-IFN as adjuvant therapy (116). In a phase Ib trial, the combination of pembrolizumab and peg-IFN revealed limited antineoplastic activity for advanced melanoma (117). However, IFN-α is still under clinical trials, in combination with targeted therapy (NCT01943422) and other immunotherapies (NCT00002882, NCT01729663). Before applying INF-α, some biomarkers have to be studied, such as STAT5 (118).

### Vaccine and Oncolytic Virus Therapy

Tumor vaccine is one of the hot topics in recent years and its principle is tumor antigen such as tumor cells, tumor-associated proteins or peptides, and genes expressing tumor antigens, which enhance immunogenicity and activate host's immune system to generate antitumor response. Generally, tumor vaccines can be categorized into four main areas: (1) recombinant full-length proteins or short peptides of TAAs, which rely on the uptake and presentation by APCs, and are recognized and bond by the molecular on the surface of APCs; (2) whole cell vaccines which include tumor cell vaccines that inactivate autologous or allogeneic tumor cells sometimes genetically modified and DCs-based vaccines which include autologous DCs loaded *ex vivo* with TAAs or related fusion proteins; (3) vectors, DNA or RNA or virus which encode TAAs; (4) whole tumor lysate or tumor cell lysate. Although objective results have been received in some cancers, overall tumor vaccine has been struggling to improve the survival. Potential challenges may include: (1) poor antigenicity of tumor antigen; (2) lack of vaccine adjuvant; (3) targeting of weak TAAs presentation antigen; (4) heterogeneity of cancer; (5) immunosuppressive networks; (6) individual differences. But the most important factor is the insufficiency in TILs which killed tumor cells specifically to eliminate tumor (119). More recent studies have proved that expanding autologous functional TILs against neo-antigens can help eliminate tumor. Neo-antigens are encoded by somatic mutations which play an important role in the development of cancer and tumor vaccine designed to target these specific tumor neo-antigens is a prospective strategy in the treatment of cancer (120). Recent researches verified that immunogenic neo-antigens is a remarkable progress that four of six patients of metastatic melanoma were recurrence-free at 25 months with neo-antigen vaccination and other two patients who received combination with immune checkpoint blockade reached complete response after regression (121). Moreover, Sahin et al. showed that eight of thirteen patients were recurrence-free at 23 months with vaccinations and other five patients had progressing metastases at the time of vaccination, two of which had objective responses (122). Personalized immunotherapy for patients with advanced or metastatic cancer is around the corner.

Oncolytic virus is an effective regimen for the treatment of cancer not only because the anti-tumor activity which kills tumor cell directly, but also the interactions among tumor cells, virus, and immune environment. The selective replication of genetically modified oncolytic virus enables it to target tumor cells. Oncolytic virus can induce both innate immunity and adaptive immunity. The antineoplastic response can be strengthened when tumor-associated antigen or cytokine are encoded by genetically engineered virus (123). Talimogene laherparepvec (T-VEC), a genetically modified oncolytic herpes simplex type 1 virus, is currently the only oncolytic virus approved by FDA for the treatment of unresectable and advanced melanoma in 2015. In a phase III trial, melanoma patients with T-VEC produced higher durable response compared with GM-CSF only (16 vs. 2%) (124). In addition to produce GM-CSF, T-VEC can also promote the release and presentation of TAAs by killing tumor cells directly and traffic and infiltrate T cells to augment the anti-tumor response (125). In another phase Ib clinical trial, combination of T-VEC and pembrolizumab significantly improved the objective response rate (62%) and complete response rate (33%) by immune-related response criteria (irRC) comparing with pembrolizumab alone. It also proved that T-VEC augmented the antitumor immune response by effecting the tumor environment (126). A following phase Ib trial in patients with unresectable stage IIIB-IV melanoma showed that combination T-VEC with ipilimumab had greater efficacy and tolerable safety (127). A larger randomized phase III trial of T-VEC plus pembrolizumab compared with pembrolizumab alone is underway (NCT02263508). These studies indicate that combining T-VEC with immune checkpoint is a promising therapeutic strategy.

## Combination Of Targeted Therapy And Immunotherapy

**Figure 2:**
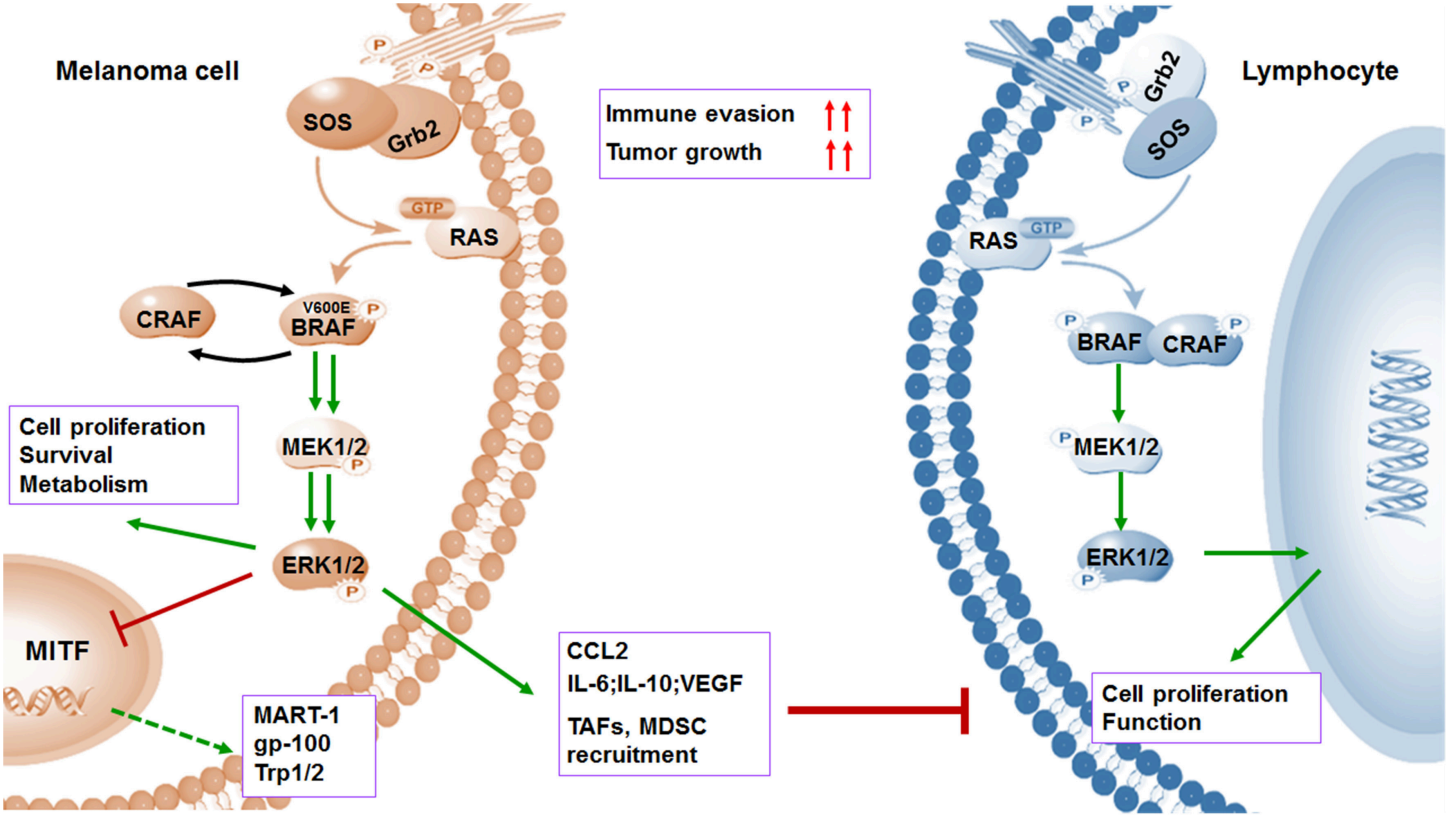
BRAF^V600E^ oncogene promotes melanoma cell proliferation and immune evasion. Mutations in BRAF oncogene cause constitutive activation of the MAPK pathway and lead to the uncontrolled proliferation of tumor cells by various mechanisms including induced anti-apoptosis, increased invasiveness, and metastatic behavior. However, activation of MAPK pathway leads to a marked reduction in tumor-associated antigens (TAAs) (MART-1, gp-100, and Trp1/2) through inhibiting transcriptional expression of MITF. Meanwhile, the activation of MAPK pathway could contribute to increased immunosuppressive regulators such as IL-6, IL-10, VEGF, IL-1, and CCL2, as well as enhanced recruitment of TAF and MDSCs. Both downregulation of antigens and upregulation of immunosuppressive factors contribute to immune evasion.

**Figure 3:**
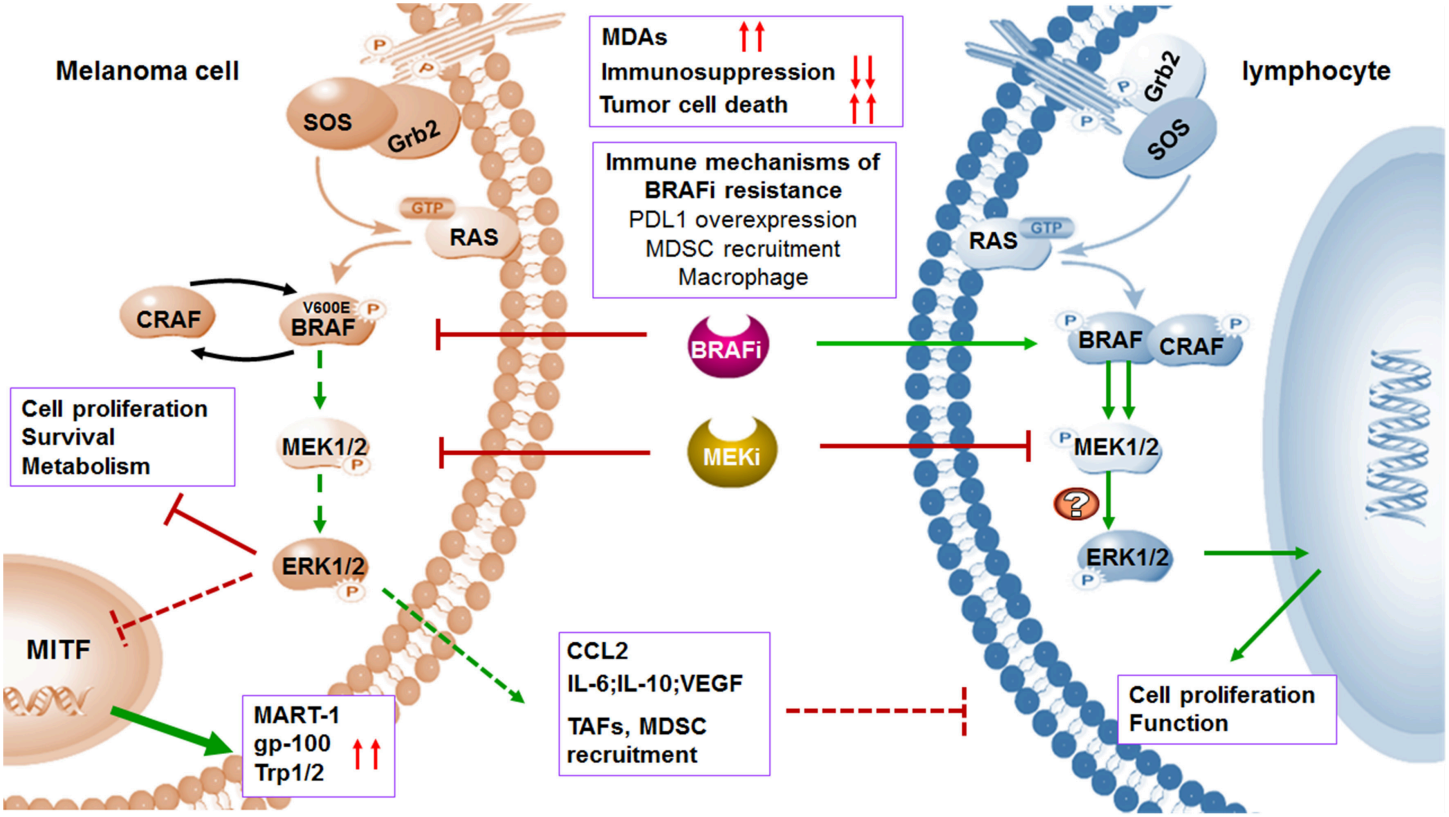
MAPK inhibitors induce melanoma cell death and regulate immune microenvironment. BRAF and MEK inhibitors induce melanoma cell death through suppression of MAPK pathway. The expression of TAAs will be increased by upregulated transcription of MITF when MAPK pathway is blocked. In addition to affecting melanoma cells, MAPK pathway blockade can also abolish the tumor immunosuppressive microenvironment including inhibition of TAFs and downregulation of immunosuppressive factors. Treatment of selective BRAF inhibitors in BRAF wild type lymphocytes leads to paradoxical activation of MAPK pathway by the transactivation of CRAF, thus promoting cell proliferation and function. Although MEK inhibitors may impair T cell function *in vitro* via MAPK pathway blockade, combination with BRAF inhibitors increased expression of antigens and suppressed immunosuppressive environment. Immune microenvironment also contributes to acquired resistance to BRAF inhibitors.

**Figure 4:**
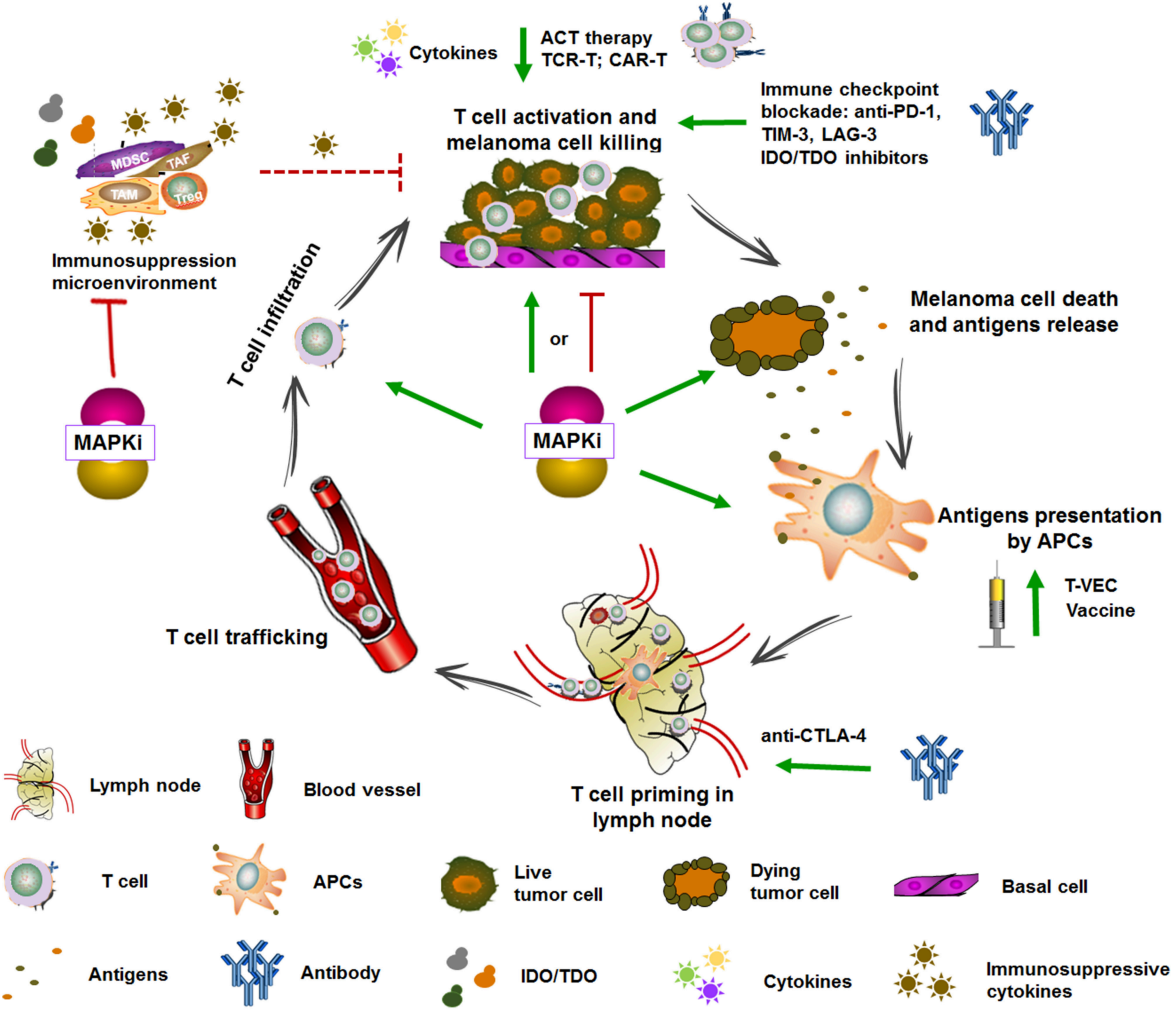
Targeted therapy and immunotherapy in the cancer-immunity cycle. The generation of antitumoral immune response is complex and involves diverse immune cells and multiple steps which include augmenting TAAs presentation, T cell priming and differentiation, enhancing the infiltration and activity of T cell, and overcoming the immune suppressive signaling. Some of immunotherapies are currently under clinical evaluation. For example, vaccine and T-VEC can promote antigens presentation, anti-CTLA-4 can promote T cell priming, cytokines, anti-PD-1, TIM3, and IDO/TDO inhibitors can promote T cell activation, and ACT therapy can directly transfer reactive and tumor-specific T cells. MAPK inhibitors complement immunotherapy through enhancing TAAs expression, promoting DCs maturation and antigens presentation, T cell infiltration into tumors, impairing immunosuppressive microenvironment, and inducing immunogenic tumor cell death. Adapted from Hughes et al. (47).

Although the response duration of immunotherapy is optimal, the rate of response is low in patients with metastatic melanoma because of the initiation of immune evasion. It occurs that cells escape from immune monitoring with multiple mechanisms, including reducing the immunogenicity, creating an immunosuppressive environment, and impairing T cell effector function. Clearly, it is impossible to achieve significant progress in OS with single therapeutic approach for the majority of patients. It has been widely reported in previous studies that oncogenic BRAF contributed to immune evasion and targeted agents such as BRAF and MEK inhibitors which effected profoundly in antigen processing and presentation, T cell priming and infiltration, and the regulation of immune microenvironment, except for the antitumor activities of targeted therapy (Figures 2–4). Therefore, combining targeted therapy with immunotherapy, especially immune checkpoint blockade, is a scientific and prominent strategy in effort to maximize therapeutic benefits and minimize toxicity. Under such circumstances, a serial of pioneer explorations have been carried out.

### Inhibition of MAPK Pathway Leads to Increase Expression of Melanocyte Differentiation Antigens (MDAs)

The processing, presentation, and recognition of melanoma antigens by APCs are important to the activation of T cells. Inadequate antigen expression may result in tumor recurrence after immunotherapy (128). Recent studies demonstrated that treating BRAF-mutant melanoma cells with BRAF inhibitors leads to significantly upregulated expression of MDAs such as MART-1, gp-100, Trp-1, and Trp-2. This treatment may also induce expression of cell surface molecule MHC I complex both in melanoma cell lines and in tumor tissues of patients with metastatic melanoma. Therefore, recognition and effector function of antigen-specific T-cells shall be intensified (129–131). Additionally, the expression of MDAs could be increased in any melanoma cells regardless of BRAF mutation status (132). The mechanism behind this is likely that the transcriptional expression of MITF and subsequent expression of MITF targets (MDAs) are increased when MAPK pathway is blocked by inhibitors. Meanwhile, it has been reported in previous studies that MITF or loss of antigen expression conferred resistance to multiple targeted agents in melanoma (129, 133). Therefore, during the progression in melanoma patients with BRAF inhibitors, the expression of melanoma antigens was dramatically decreased along with downregulation of MITF (134). However, these effects can be reversed by additional MEK inhibitors so that MEK inhibitors and BRAF inhibitors can synergize with each other (130). Furthermore, combination of BRAF inhibitors and MEK inhibitors upregulates melanoma antigens and HLA class I molecules in BRAF-mutant melanoma cell lines as demonstrated by Liu et al. (135). On the other hand, overexpression of melanoma antigens may result from the uptake and processing of apoptotic melanoma cells by antigen presenting cells and can be recognized and cross-presented to T cells (136).

### Inhibition of MAPK Pathway May Enhance the Function of DCs

Dendritic Cells are antigen processing cells which play an important role in the priming and activation of antigen-specific T cell responses. Sumimoto et al. demonstrated that BRAF oncogene leads to aberrant activation of MAPK pathway and contributes to the generation of immunosuppressive tumor microenvironment by producing immunosuppression cytokines including IL-6, IL-10, and VEGF (137). In this way, it suppresses the secretion of IL-12 and TNF-α and decreases the expression of co-stimulatory molecules such as CD80, CD83, and CD86 on DCs (138). Notably, BRAF and MEK inhibitors may reverse the suppression of cytokines released by DCs in BRAF^V600E^ mutant cell lines to enhance DCs function, which does not work in BRAF^WT^ cell lines. Further researches demonstrated that BRAF inhibitors have none effect on DCs function even if at high dosage, while the effects on DCs function with MEK inhibitors were controversial and needed to be validated *in vivo* (138, 139). Interestingly, Vella et al. showed that the combination of MEK and BRAF inhibitors promoted DCs maturation while reduced antigens cross-presentation (140).

### Inhibition of MAPK Pathway May Strengthen the Function of Natural Killer Cells and T Cells

The MAPK signaling pathway and BRAF inhibition not only change DCs function, but also affect the activities of natural killer (NK) cells and T cells priming, infiltration and function. NK cells are powerful cytotoxic lymphocytes that spontaneously lyse tumor cells, and mediate innate and adaptive antitumor immune responses by producing cytokines. In BRAF^WT^ cells, selective BRAF inhibitors generate paradoxical activation of MAPK pathway via the transactivation of CRAF (141, 142). Accordingly, PLX4720 (a BRAF inhibitor) treatment directly strengthens the ERK phosphorylation in murine and human NK cells. Meanwhile, proliferation and CD69 expression of NK cells are increased in the context of IL-2, which is important for NK cells to reinforce the antitumor activity of BRAF inhibitor by perforin-dependent pathway in a BRAF-mutant melanoma mouse model (143). Researchers also showed that depletion of NK cells by antibodies failed in reducing the incidence of metastases in lung cancers, but reduction was observed in CD4 and CD8 depleted mice with BRAF inhibition. Interestingly, Schilling et al. found that the number of NK cells increased after treatment with BRAF inhibitor in melanoma patients (144). However, recent researches suggested that BRAF inhibition treatment decreased the expression of NKG2D ligands (MICA and ULBP2) and increased the expression of NKp30 ligand B7-H6. At the same time, it induced the expression of inhibitory molecules such as HLA class I and HLA-E which suppressed the antitumor activity of NK cells. This shift in the balance between inhibitory and stimulatory NK cells ligands on BRAF^MUT^ melanoma cell lines caused by BRAF inhibition regulated NK cells function (145). The combination of targeted therapy and immunotherapy synergize only when the targeted-agents are nontoxic to systemic immunity. Effector T cells play a dominant role in immune response. According to the previous studies, selective BRAF inhibitors (PLX4032 and PLX4720) do not affect the viability and functionality of T cells (146) and MEK inhibitor-impaired T cells *in vitro* (129). However, trametinib did no adversely affect the function of T cells *in vivo* (147). Moreover, combination of trametinib and dabrafenib may regulate tumor microenvironment with pmel-1 adoptive cell transfer (ACT) through decreasing Tregs and macrophage infiltration which prevent effector T cells from entering tumor cells. Subsequent studies have shown that pan-BRAF inhibitor (BMS-908662) may directly activate T cells in a dose-dependent manner by paradoxical ERK signaling activation in BRAF^WT^ cells (148). Previous study also showed that TILs from mice which were treated with vemurafenib were higher functionally activated. And when they were re-exposed with antigen, the secretion of immune stimulatory cytokine IFN-γ was increased (136). Evidences from preclinical studies and early clinical trials showed MAPK inhibition soon improved the infiltration and activation of intratumoral antigen-specific CD8^+^ T cells (130). This may be a result of enhancement of melanoma antigens with BRAF treatment. Importantly, previous studies suggested that BRAF inhibition significantly increased the infiltration and activation of T cells within 10–14 days (149). However, in addition to overexpression of markers of T cell cytotoxicity including perforin and granzyme B, T cell exhaustion markers PD-1 and TIM3, and PD-L1 expression in immune microenvironment were also increased with BRAF inhibition (130). These are powerful evidence that additional immune checkpoint blockade may enhance antitumor activity with BRAF inhibition. Unfortunately, favorable immune responses to MAPK-targeted therapy are prompt but transient, and it no longer responses at the progression of melanoma. More specifically, the expression of antigens is terminated and the number of CD8^+^ T cells is significantly decreased than that at therapy initiation. Therefore, additional researches are in need to determine the appropriate timing and sequence of therapy in order to further promote tumor elimination with combinatorial therapy. Currently, some pertinent clinical trials are underway (Table 1).

**Table 1:** Clinical trials of combination of MAPK pathway targeted therapy and immunotherapy in advanced or metastatic melanoma.

**NCT number**	**Targeted therapy**	**Immunotherapy**	**Status**	**Trial Phase**	**Scheduling**
NCT01400451 (150)	Vemurafenib	Ipilimumab	Terminated	Phase I	Concurrent
NCT01673854 (151)	Vemurafenib	Ipilimumab	Completed	Phase II	Sequential
NCT02200562	Dabrafenib	Ipilimumab	Terminated	Phase I	Concurrent
NCT01767454 (152)	Dabrafenib or dabrafenib + trametinib	Ipilimumab	Completed	Phase I	Concurrent
NCT01245556	BMS-908662	Ipilimumab	Completed	Phase I	Concurrent
NCT01656642	Vemurafenib or vemurafenib + cobimetinib	Atezolizumab	Active, not recruiting	Phase I	Concurrent
NCT03178851	Cobimetinib	Atezolizumab	Recruiting	Phase I	Concurrent
NCT02027961	Dabrafenib or trametinib or dabrafenib + trametinib	MEDI4736	Completed	Phase I/II	Concurrent sequential
NCT02130466	Trametinib + dabrafenib	Pembrolizumab	Recruiting	Phase I/II	Concurrent
NCT02357732	Dabrafenib or trametinib or dabrafenib + trametinib	Nivolumab	Withdrawn	Phase I	Concurrent
NCT02818023	Vemurafenib + cobimetinib	Pembrolizumab	Recruiting	Phase I	Concurrent
NCT02858921	Dabrafenib + trametinib	Pembrolizumab	Recruiting	Phase II	Concurrent; Sequential
NCT02625337	Dabrafenib + trametinib	Pembrolizumab	Recruiting	Phase II	Concurrent
NCT02967692	Dabrafenib + Trametinib	PDR001	Recruiting	Phase III	Concurrent
NCT02902042	Encorafenib + binimetinib	Pembrolizumab	Recruiting	Phase I/II	Concurrent
NCT03554083	Cobimetinib or vemurafenib + cobimetinib	Atezolizumab	Recruiting	Phase II	Concurrent
NCT02902029	Cobimetinib + vemurafenib	Atezolizumab	Recruiting	Phase II	Sequential
NCT03235245	Encorafenib + binimetinib	Ipilimumab + nivolumab	Not yet recruiting	Phase II	Sequential
NCT02968303	Vemurafenib + cobimetinib	Ipilimumab + nivolumab	Recruiting	Phase II	Sequential
NCT01940809	Dabrafenib or trametinib or dabrafenib + trametinib	Ipilimumab or nivolumab or ipilimumab + nivolumab	Active, not recruiting	Phase I	Sequential
NCT02224781	Dabrafenib + trametinib	Ipilimumab + nivolumab	Recruiting	Phase III	Sequential
NCT02631447	LGX818 + MEK162	Ipilimumab + nivolumab	Recruiting	Phase II	Sequential
NCT01603212	Vemurafenib	IL-2 + Interferon α-2b	Completed	Phase I/II	Concurrent
NCT01754376 (153)	Vemurafenib	Aldesleukin	Terminated	Phase II	Concurrent
NCT01683188 (154)	vemurafenib	High-does IL-2	Terminated	Phase IV	Concurrent
NCT01943422	vemurafenib	High-dose Interferon α-2b	Completed	Phase I	Concurrent
NCT01959633	Vemurafenib	PEG-interferon	Recruiting	Phase I/II	Concurrent
NCT02354690	Vemurafenib	Lymphodepleting chemotherapy + TILs+ IL-2	Active, not recruiting	Phase I/II	Concurrent
NCT01659151	Vemurafenib	Lymphodepletion+ ACT with TIL Infusion +IL-2	Active, not recruiting	Phase II	Concurrent
NCT01585415 (155)	Vemurafenib	Aldesleukin + Young TIL	Terminated	Phase I	Sequential
NCT02382549	Dabrafenib + trametinib	6MHP (6 melanoma helper peptide vaccine)	Recruiting	Phase I/II	Concurrent
NCT03088176	Dabrafenib + trametinib	Talimogene Laherparepvec	Recruiting	Phase I	Concurrent

### Inhibition of MAPK Pathway Impairs Hostile Tumor Microenvironment

Apart from influencing immune cells, MAPK pathway and BRAF blockade may also destruct the tumor immunosuppressive microenvironment via inhibition of tumor-associated fibroblasts (TAFs), downregulation of immunosuppressive factors such as chemokine CCL2, VEGF, IL-10, IL-6, MDSCs and Tregs, and upregulation of tumor-associated macrophages (TAMs) (137). As confirmed by Steinberg et al. it significantly decreased Tregs and depleted intratumoral MDSCs within the tumor microenvironment, and thus promoted tumor eradication in BRAF/PTEN genetically engineered mouse model treated with BRAF inhibitor (PLX4032). The authors also proved that this antitumor response was CD8^+^ T cell-dependent (156), and that PLX4032 also decreased CCL2 expression in tumor microenvironment in this model (157). Vascular endothelial growth factor (VEGF), a potential immunosuppressive proangiogenic factor, may block the maturation and differentiation of DCs and induce expression of MDSCs (158). MEK inhibitor (U0126) or RNAi has been proved to reduce the expression of angiogenic factors such as VEGF, as well as decrease immunosuppressive cytokines including IL-6 and IL-10 (137). Abrogating TAFs which was mediated by IL-1 in the stroma is another mechanism of suppression of immunosuppressive tumor microenvironment with BRAF inhibition (159). Unfortunately, MAPK blockade (BRAF and MEK inhibitors) significantly increased the expression of TNF-α and the number of TAMs including M1/M2 macrophages in patients biopsies (160), which mainly played an inhibitory role even though M1 macrophage might inhibit tumor growth.

### Immune Evasion Contributes to Resistance to MAPK Inhibitors

Immune microenvironment also contributes to acquired resistance to BRAF inhibitors except for an important role in the response of tumors to BRAF targeted therapy. As described above, BRAF inhibitors failed to eradicate melanoma due to immune evasion to some extent. Several studies showed expression of inhibitory ligand PD-L1 was dramatically increased with treatment of BRAF inhibitors in melanoma cell lines and patient-derived biopsies with metastatic melanoma (130). Jiang et al. proved that overexpression of PD-L1 contributes to acquired clinical resistance to BRAF inhibitors in melanoma patients through MAPK reactivation and strengthen of interaction of c-JUN and STAT3. However, additional MEK inhibitors may downregulate the expression of PD-L1 and promote tumor cell apoptosis (161). Recent studies proved that overexpression of PD-L1 may be a result of constitutive YAP activation and enhanced transcription of CD274 gene (162). These studies suggested that the combination of MAPK inhibition and PD-L1 blockade may promote melanoma elimination. Macrophage-derived TNF-α is also involved in the resistance to BRAF inhibitors (160). Moreover, restoration of MDSCs recruitment is also an indicator of resistance which depends on MAPK activation. Therefore, MEK inhibitor reduces intratumoral MDSC accumulation and inhibits proliferation of BRAF inhibitor-resistant cells. Furthermore, the resistance to BRAF inhibitors might be exempted with a combination therapy of immune checkpoint blockade (anti-CTLA-4 and anti-PD-1), MDSC deletion and CCR2 antagonist (163).

## Preclinical Studies of Combination of Immunotherapy and Targeted Therapy

To date, several studies have focused on the synergy in combined regimen of targeted therapy and immunotherapy in mouse models. Callahan et al. found that BMS908662 (a BRAF inhibitor) could enhance the antitumor activity of CTLA-4 blockade in mice model by significantly promoting the expansion of antigen-specific CD8^+^ T cells (148). Similar antitumor response has been observed with the combination of BRAF inhibition and PD-1 pathway blockade through increasing number and function of tumor-infiltrating T cells (164). If PD-L1 expression is increased in BRAF inhibition combined with MEK inhibition, triple combination of PD-L1, BRAF, and MEK blockade will be ideal in antitumor effects (147). While the combination of targeted therapy and immune checkpoint blockade resulted in fantastic antitumor activity *in vivo*, additional immune stimulatory agents should be considered to improve this response (165). Yang Liu et al. showed that MEK inhibitor upregulated expression of TIM-3 and combination of trametinib with TIM-3 blockade promoted tumor elimination (166).

Apart from the combination of immune checkpoint blockade and MAPK inhibitors, several other immunomodulatory molecules may also cooperate with targeted therapy in preclinical models. In the study of Koya et al. the combined treatment of pmel or OVA TCR gene-engineered ACT therapy plus vemurafenib has significantly improved antitumor activity by enhancing effector function of intratumoral T cells in SMA or SM1-OVA model, which provided strong theoretical support for the combinatorial regimen of targeted therapy and ACT therapy for the treatment of patient with metastatic melanoma harboring BRAF mutation (136).

## Clinical Trials of Combination of Immunotherapy and Targeted Therapy

Based on the concrete proof that combination of targeted therapy and immunotherapy is a promising strategy, plenty of combinatorial regimens are being evaluated in clinical trials. Up to now, trials mainly focused on BRAF inhibitions with or without MEK inhibitions combined with immune checkpoint blockade, cytokines such as IL-2, and IFN α-2b/peg-IFN, vaccine, and adoptive T cell therapy (Table 1).

The combination of BRAF inhibitor with CTLA-4 blockade has been tested in multiple clinical trials. Though some trials have shown encouraging results, no prospective data of appropriate treatments such as what and how to combine is available. A phase I trial (NCT01400451) assessing the combination of ipilimumab and vemurafenib has been terminated due to unexpected grade 2/3 hepatotoxicity in patients with BRAF^V600E^-mutant metastatic melanoma (150). However, a phase II study (NCT01673854) of vemurafenib followed by ipilimumab has not shown severe hepatotoxicity but reported a grade 3/4 skin adverse event (151). It is possible given that BRAF inhibition can maintain a favorable immune microenvironment within 10–14 days before additional anti-CTLA-4 antibodies. Combination of BMS-908662 (pan-RAF inhibitor) with ipilimumab is also being tested in a phase I trial (NCT01245556). Unfortunately, a phase I/II trial of combination of dabrafenib with ipilimumab in stage III/IV melanoma has been terminated because ipilimumab withdrew support (NCT02200562). Moreover, severe gastrointestinal toxicity has been seen in melanoma patients who administrated triple combination of trametinib, dabrafenib, and ipilimumab, despite an absence of hepatic toxicities (NCT01767454) (152). These clinical trials demonstrated that combinatorial therapy has facilitated an excessive severe toxicity. Therefore, how to design and conduct a combinatorial regimen which intensifies antitumor effectiveness without increasing additional toxicities is a crucial issue. In addition to anti-CTLA-4 antibodies, several anti-PD-1/PD-L1 antibodies were tested in combination with BRAF/MEK inhibitors in metastatic melanoma (NCT01656642, NCT02130466, and NCT02818023). Given toxicity of PD-1/PD-L1 blockade is drastically lower than that of anti-CTLA-4 antibodies in previous studies (71), it may achieve outstanding outcomes in melanoma patients when combined with MAPK inhibition. A recent study demonstrated that combination of MAPK inhibition with anti-PD-L1 antibody (MEDI4736) received great disease control rates and was well-tolerated with no additional toxicities beyond what would be expected with monotherapy (167). Furthermore, several clinical trials are underway for the sake of exploring appropriate timing and sequence of combinatorial therapy in metastatic melanoma (Table 1) because proper timing and sequence is another vital consideration before administrating combinatorial regimens. A randomized phase III study (NCT02224781) is going to evaluate which regimen is better in treatment of patients with stage III/IV unresectable or metastatic melanoma harboring BRAF^V600E/K^ mutation, MEK and BRAF inhibition (trametinib plus dabrafenib) followed by PD-1 and CTLA-4 blockade (nivolumab plus ipilimumab) or PD-1 and CTLA-4 blockade (nivolumab plus ipilimumab) followed by MEK and BRAF inhibition (trametinib plus dabrafenib).

Currently, four open clinical trials have been or are evaluating the combination of vemurafenib with cytokines in advanced or metastatic melanoma. In a phase II study (NCT01754376), high response rates (overall response rate was 83.3% at 6 weeks) were observed in patients with unresectable stage III or IV melanoma harboring BRAF^V600E^ mutation treated with vemurafenib and HD IL-2 (153). Although all patients experienced grade 3 toxicity, they were successfully managed with supportive care. It was also proved that cfDNA may act as a biomarker for treatment response and disease progress since the levels of cfDNA were associated with response in patients treated with combinatorial regimen. Contribution of BRAF inhibitor to antitumor activity through increasing expression of antigens and enhancing infiltration and effector function of specific-CD8^+^ T cell was demonstrated in this study. However, these effects may be attenuated by concurrent increase of Treg with HD IL-2 administration, suggesting a potential mechanism of resistance to this treatment approach. Regretfully, a phase IV study including two cohorts (cohort 1: vemurafenib was administrated for 6 weeks prior to HD IL-2 treatment; cohort 2: vemurafenib was administrated for 7 to 18 weeks with stable or responding disease prior to starting HD IL-2) demonstrated that combination of HD IL-2 with vemurafenib did not show the synergy in treatment of BRAF-mutated metastatic melanoma except expected response with either agent alone (154). In addition to IL-2, combinations of vemurafenib with IFN α-2b or peg-IFN were tested in patients with metastatic melanoma (NCT01943422, NCT01959633). MAPK pathway activation can promote the degradation of IFNAR1, a subunit of IFN receptor, and BRAF inhibition was able to restore IFNAR1 expression in cell lines and patient-derived biopsies. Furthermore, the antitumor activity of BRAF inhibition and IFN-α combination *in vitro* and *in vivo* were also confirmed by Francesco Sabbatino et al., which provided a strong rational evidence for combination of vemurafenib and IFN-α (168).

Surprisingly, two patients with stage IIIB/C metastatic melanoma benefited from T-VEC after disease progression with multiple therapies including BRAF/+MEK inhibition, immune checkpoint blockade, and GM-CSF (169). Patient 1 received a complete response at 23 weeks, patient 2 continued to accept therapy after 60 weeks whose biopsy showed noticeable specific-CD8^+^ and CD4^+^ T cells infiltration after treatment for 1 year. Moreover, there were no new safety signals except expected adverse effects treated with T-VEC. Another phase I trial is about to test the effect of dabrafenib, trametinib combined with T-VEC (NCT03088176). A trial on the regimen of dabrafenib, trametinib combined with 6MHP (6 melanoma helper peptide vaccine) is also ongoing (NCT02382549).

Recently, the first study showing successful combination of TILs with vemurafenib for the treatment of metastatic melanoma has been presented (155). In this phase I trial (NCT01585415), patients were taking vemurafenib for 2 weeks prior to the resection of metastatic tumor for growth of TILs. Then the patients received a lymphodepleting pre-conditioning regimen, infusion of autologous TILs with HD IL-2 administration and vemurafenib was restarted at the time of TIL infusion. Treatment achieved well-objective response (64%), including 18% of complete regressions in metastatic melanoma which confirmed that the combination of ACT with vemurafenib was safe and feasible. Given resistance to BRAF inhibition, ACT, BRAF inhibitors combined with MEK inhibitors may be a profound regimen. Additionally, another two phase II clinical trials are underway (NCT02354690, NCT01659151).

## Conclusion and Future Perspectives

Although oncogene-targeted therapy is an effective regimen for the majority of metastatic melanoma patients, the response is not durable due to promptly developed acquired resistance. On the contrary, immunotherapy enables long-term disease control, but the response rate is limited. On the one hand, more and more evidence indicated that targeted agents synergize the function of immune cells and immune microenvironment endorsing the rationale of combinatorial therapy. The potential molecular mechanisms included: (1) promotion of melanocyte differentiation antigens expression; (2) agitation of T cell infiltration into tumor microenvironment; and (3) abrogation of immunosuppressive tumor microenvironment (170). Emerging preclinical and clinical studies also proved the advantage of synergizing oncogene-targeted therapy and immunotherapy (164, 167). On the other hand, concomitant administration of targeted therapy with immunotherapy has generated serious adverse events. For example, the clinical trial of ipilimumab in combination with vemurafenib in advanced melanoma were suspended due to hepatotoxicity (150). It is also important to investigate proper sequencing of combination targeted therapy (BRAF or MEK inhibitor) with immune checkpoint blockade (anti-CTLA or anti-PD-1/PD-L1 antibody), because patients treated with targeted agents may display distinct immune-compatibility: either be more sensitive or be more tolerant. Apart from toxicity, proper sequence, and timing of therapies should be considered and accessed when the combinatorial regimens are designed. As discussed before (171), specific biomarkers or predictors of response and adverse events may be important to achieve more precise personal treatment. Although MEK inhibitors can create favorable tumor microenvironment, they may impair the function of antigen-specific T cells by inhibiting physiological MAPK pathway. Therefore, how to magnify synergy of combinatorial regimens through avoiding small molecular inhibitors induced T cell toxicity is another challenge. In order to minimize toxicity of combination targeted therapy with immunotherapy, novel drugs and innovative combinatorial strategies need to be further explored in the further. Better understanding the complex interference between targeted therapy and immunotherapy will be helpful to develop more effective agents and to design better combinatorial regimens.

For the sake of a successful precision medicine, it is highly desired to identify and characterize the biomarkers that predict response or adverse events with targeted or immunotherapeutic drugs. Initially, we need to determine which subpopulation of patients are likely to benefit from targeted therapy or immunotherapy, in other words, what biomarkers can be used to predict the effect of drugs before treatment. For MAPK targeted therapy, melanoma should be addicted to MAPK pathway without aberrant activation of alternate growth pathways such as EGFR, KIT, and AKT mutation. In some of BRAF-resistant melanoma cells, PD-L1 expression is upregulated, which may predict that the combining with anti-PD-L1 is a rational regimen. In addition to higher expression of PD-L1, increasing melanoma antigens expression, T-cell infiltration into tumor, or release of cytokines (IL-12, IFN γ) are likely to predict that combination with immune checkpoint blockade is a rational strategy. Moreover, tumor mutation burden (TMB) and blood tumor mutation burden (bTMB), diversity of HLA, deficient mismatch repair (dMMR) and MSI-H, and DNA methylation may be the biomarkers which will response to immune checkpoint blockade therapy (172–175). Deutsch et al. provided a promising imaging biomarker (CT images) to assess CD8^+^ T cell infiltration and response to immune checkpoint blockade therapy. It can also predict clinical outcomes for patients (176). Additionally, if patients are under treatment, detecting certain biomarkers that can accurately evaluate the effects is also important to individualized treatment. Medical imaging has been widely applied to evaluate response with drugs due to the intuitive description of the tumors size. However, it is possible to present false-positive in treatment because immunotherapy might recruit immune cells surrounding tumor and makes it look larger. Therefore, biomarkers which are relatively easy to obtain are in urgent need. Previous researches proved that ctDNA from peripheral blood, IL-8 concentration in serum, expression of soluble CD25, and expression of CD39 on immune cells may be potential biomarkers to monitor the effects under treatment (177–181).

Better understanding the mechanisms of drugs that inhibit targets will be helpful to develop more effective agents, such as drugs against novel targets, the next-generation new drugs. For example, numerous studies suggest that ipilimumab mediated antitumor activity is due to the depletion of Tregs in tumor environment rather than blockade of B7-CTLA-4 interaction and this mechanism depends on Fc receptor on host cells (58). Du et al. also proved that blocking B7-CTLA-4 interaction impaired neither the safety nor efficacy of antibodies against CTLA-4, which provided novel insights for clinical development of a safer and more efficient CTLA-4-targeting reagents to eradicate cancer (182). BRAF^V600E^ inhibitors are another potential direction which may paradoxically activate rather than suppress MAPK signaling pathway in BRAF wildtype immune cells. As the development of genomic, transcriptomic, proteomic, and epigenetic technologies, the prospects of novel drugs targeting BRAF mutation or new targets similar to BRAF mutation are very optimistic.

## Author Contributions

CY conceptualized, wrote, and edited the manuscript. CY, XL, and MZ created the figures. XL, JY, MZ, HJ, XM, and HS provided feedback and reviewed the manuscript.

### Conflict of Interest Statement

The authors declare that the research was conducted in the absence of any commercial or financial relationships that could be construed as a potential conflict of interest.
